# Semi-Fluorinated Di and Triblock Copolymer Nano-Objects Prepared via RAFT Alcoholic Dispersion Polymerization (PISA)

**DOI:** 10.3390/polym13152502

**Published:** 2021-07-29

**Authors:** Gregoire Desnos, Adrien Rubio, Chaimaa Gomri, Mathias Gravelle, Vincent Ladmiral, Mona Semsarilar

**Affiliations:** 1 Institut Européen des Membranes, IEM, UMR 5635, Univ Montpellier, ENSCM, CNRS, Montpellier, France; gregoire.desnos@outlook.fr (G.D.); rubiioadrien@gmail.com (A.R.); chiamaa.gomri@umontpellier.fr (C.G.); mathias.gravelle@enscm.fr (M.G.); 2 ICGM, Univ Montpellier, CNRS, ENSCM, Montpellier, France; vincent.ladmiral@enscm.fr

**Keywords:** semi-fluorinated, RAFT, PISA, dispersion polymerization, self-assembly

## Abstract

A set of well-defined amphiphilic, semi-fluorinated di and triblock copolymers were synthesized via polymerization-induced self-assembly (PISA) under alcoholic dispersion polymerization conditions. This study investigates the influence of the length, nature and position of the solvophobic semi-fluorinated block. A poly(*N*,*N*-dimethylaminoethyl methacrylate) was used as the stabilizing block to prepare the di and tri block copolymer nano-objects via reversible addition-fragmentation chain transfer (RAFT) controlled dispersion polymerization at 70 °C in ethanol. Benzylmethacrylate (BzMA) and semi-fluorinated methacrylates and acrylates with 7 (heptafluorobutyl methacrylate (HFBMA)), 13 (heneicosafluorododecyl methacrylate (HCFDDMA)) and 21 (tridecafluorooctyl acrylate (TDFOA)) fluorine atoms were used as monomers for the core-forming blocks. The RAFT polymerization of these semi-fluorinated monomers was monitored by SEC and ^1^H NMR. The evolution of the self-assembled morphologies was investigated by transmission electron microscopy (TEM). The results demonstrate that the order of the blocks and the number of fluorine atoms influence the microphase segregation of the core-forming blocks and the final morphology of the nano-objects.

## 1. Introduction

Colloidaly stable nano-objects from self-assembly in solution of amphiphilic block copolymers have been the subject of considerable research over the last 20 years. This is mainly due to the advances in the field of controlled polymerization, the wide range of possible functional monomers as well as the numerous potential fields of application for such particles, from drug delivery to nanosensors or filtration membranes, for example. The morphology and size of nano-objects prepared by self-assembly of block copolymers depend on various factors of which the most important ones are the relative length of the blocks, the interaction between the solvophilic block chains and the interfacial energy of the solvophilic−solvophobic interface. To control the morphology of the nano-objects, parameters such as block ratio, solvent nature and composition, block rigidity and concentration have been studied [1,2,3]. Traditionally self-assembly of block copolymers are conducted in dilute solution after polymerization and isolation of the pure block copolymers. The main limitation of this approach is that it involves several labor-intensive steps, uses low concentration and is difficult to repeat or reproduce [4,5]. The recent polymerization-induced self-assembly (PISA) method is an attractive alternative for the production of block copolymer nano-objects at high concentration [6,7,8,9,10,11]. In a RAFT-mediated PISA process, a solvophilic macromolecular chain transfer agent (macroCTA) is chain extended with solvophobic block. As the chain extension proceeds the second block become insoluble in the media and triggers the formation of nanoparticles at a certain critical degree of polymerization [12,13,14,15]. These nanoparticles are usually kinetically trapped due to low chain mobility of the core-forming (solvophobic) block [16,17]. So far the PISA approach has proven to be a reliable method to synthesize a wide range of higher order morphologies as pure phase and at high concentrations (up to 50% solids content) [3,10].

Semi-fluorinated (meth)acrylates are monomers that contain CF moieties as side groups. The presence of fluorine atoms endows them with remarkable properties such as high hydrophobicity and lipophobicity, crystallinity (if the side chain is perfluorinated and sufficiently long) and low refractive index [18,19,20,21]. They can polymerize via controlled radical polymerization techniques to afford well-defined copolymers [22,23,24]. These properties make them good candidates for many applications in self-assembly, MRI imaging, coatings or membranes, to name a few [25,26,27].

In 2014, Armes and coworkers reported the first use of a semi-fluorinated monomer in a PISA formulation [12]. They prepared ABC triblock copolymer vesicles via seeded dispersion polymerization of trifluoroethyl methacrylate (TFEMA) in ethanol with PMAA-*b*-PBzMA as the macro-CTA (MAA and BzMA stand for methacrylic acid and benzyl methacrylate, respectively). This chain extension produced a range of remarkably complex semi-fluorinated triblock copolymer morphologies, with internal phase separation driven by the enthalpic incompatibility between the PBzMA and PTFEMA core-forming blocks. However, PTFEMA does not contain enough fluorine atoms to completely phase separate from PBzMA. Later Yuan and co-workers reported in several studies that if the fluorine content of the core-forming block was increased, phase separation took place and that non-classical morphologies could be obtained [28,29,30,31]. They demonstrated this with semi-fluorinated methacrylates bearing 9, 13 and 17 fluorine atoms. They prepared ABC triblock copolymer nanoobjects via RAFT dispersion polymerization in ethanol, using a poly(*N*,*N*-dimethylaminoethyl methacrylate) (PDMA) macro-CTA, and PBzMA and poly(2-perfluorohexylethyl methacrylate) (PFHEMA) as the core-forming blocks. Varying the degrees of polymerization of the PBzMA and PFHEMA core-forming blocks resulted in the formation of multicompartment micelles (MCMs) [30,31]. In another article [29], they replaced PFHEMA with poly[2-(perfluorooctyl)ethyl methacrylate] (PFOEMA) and looked into controlling the morphology via adjusting the hydrophilic-hydrophobic block ratios. Their systematic study led to the formation of spheroids, cylinders and spherical MCMs through the interplay of the inter-chain repulsion of PDMA, the LC anisotropic ordering, and the microphase segregation between PBzMA and PFOEMA.

The present work presents the synthesis of AB and ABC block copolymers containing one semi-fluorinated block via RAFT dispersion polymerization in ethanol. A poly(*N*,*N*-dimethylaminoethyl methacrylate) (PDMA) was used as the stabilizing block while the core forming blocks were poly(benzyl methacrylate) (PBzMA) along with a semi-fluorinated block; poly(heptafluorobutyl methacrylate) (PHFBMA) or poly(heneicosafluorododecyl methacrylate) (PHCFDDMA) or poly(tridecafluorooctyl acrylate) (PDFOA). The degrees of polymerization of the core-forming blocks and their order in the block sequence along with the number of fluorine atoms (7, 13 and 21) in the semi-fluorinated hydrophobic block was varied and the effects of these parameters on the final morphologies and phase segregation were studied.

## 2. Experimental

### 2.1. Materials

All reagents were purchased from Sigma-Aldrich France (Saint Quentin Fallavier) and used as received unless otherwise noted. Recrystallized 2,2′-azobisisobutyronitrile (AIBN) was used as the initiator. Benzyl methacrylate was passed through an inhibitor removal column (also purchased from Sigma) prior to use. 4-Cyano-4-(2-phenylethanesulfanylthiocarbonyl) sulfanylpentanoic acid (PETTC) was synthesized as reported previously [32]. CDCl_3_ and CD2Cl2 were purchased from Eurisotop, Saint Aubin, France. 3,3,4,4,5,5,6,6,7,7,8,8,8-Tridecafluorooctyl acrylate (TDFOA) and 3,3,4,4,5,5,6,6,7,7,8,8,9,9,10,10,11,11,12,12,12-heneicosafluorododecyl methacrylate (HCFDDMA) was kindly donated by Atofina, France (Colombes).

#### 2.1.1. Synthesis of Poly(2-(N,N,dimethylamino)ethyl Methacrylate) (PDMA) Macro-CTA

A round-bottomed flask was charged with 2-(*N*,*N*,dimethylamino) ethyl methacrylate (DMA; 10.0 g, 63.6 mmol), PETTC (432.1 mg, 1.27 mmol; target DP = 50), ACVA (35.4 mg, 0.127 mmol; PETTC/ACVA = 10) and THF (10.0 g). The sealed reaction vessel was purged by bubbling nitrogen into the reaction mixture and placed in a pre-heated oil bath at 70 °C for 8 h. The resulting polymer (monomer conversion = 71%; *M_n_* = 6100 g mol^−1^, *Đ* = 1.16) was purified by precipitation into excess hexane. The mean degree of polymerisation (DP) of this PDMA macro-CTA was calculated to be 39 using ^1^H NMR spectroscopy (in CDCl_3_ or CD_2_Cl_2_) by comparing the integrated signals corresponding to the aromatic protons at 7.2–7.4 ppm with those of the methacrylic polymer backbone at 0.4–2.5 ppm.

#### 2.1.2. Synthesis of Poly(2-(N,N,dimethylamino)ethyl Methacrylate)-poly(benzyl methacrylate) (PDMA-PBzMA) Diblock Copolymer Particles via RAFT Dispersion Polymerisation in Ethanol

In a typical RAFT synthesis in dispersion conducted at 15% *w*/*w* total solids: BzMA (0.2 g, 1.14 mmol; target DP = 70), AIBN (0.5 mg, 0.003 mmol; PETTC/AIBN = 5), PDMA_39_ macro-CTA (99.4 mg, 0.016 mmol) was dissolved in 1.7 g of ethanol. The reaction mixture was placed in a round-bottomed flask sealed with a ruber septum, cooled down using an ice bath, purged with nitrogen gas for 5 min and then placed in a pre-heated oil bath at 70 °C for 24 h. The final monomer conversion was determined by ^1^H NMR analysis in CDCl_3_ or CD_2_Cl_2_ by integrating the PBzMA peak (CH_2_) at 4.9 ppm to the BzMA vinyl peaks (CH_2_) at 5.2 and 5.4 ppm.

In other PDMA-PBzMA diblock copolymer syntheses, the mean DP of the PBzMA block was varied by adjusting the amount of BzMA. PDMA-PHFBMA and PDMA-PHCFDDMA diblock copolymers were synthesized following the same procedure by replacing BzMA with HFBMA or PHCFDDMA.

#### 2.1.3. Synthesis of Poly(2-(N,N,dimethylamino)ethyl Methacrylate)-poly(benzyl methacrylate)-poly(heptafluorobutyl methacrylate) (PDMA-PBzMA-PHFBMA) Triblock Copolymer Particles Was Performed via RAFT Dispersion Polymerisation in Ethanol

In a typical RAFT synthesis of a triblock, HFBMA (0.217 g, 0.81 mmol; target DP = 50) and AIBN (0.5 mg, 0.003 mmol; PETTC/AIBN = 5) were purged with nitrogen in a vial. This mixture was then injected to the reaction mixture containing the PDMA-PBzMA (after 100% conversion of BzMA confirmed by NMR; See Section 2.1.2 for details). The reaction mixture was left at 70 °C for an additional 24 h. The final monomer conversion was determined by ^1^H NMR analysis in CDCl_3_ or CD_2_Cl_2_ by integrating the PHFBMA peak (CH_2_) at 4.8 ppm to HFBMAvinyl peaks (CH_2_) at 5.7 and 6.2 ppm.

The same procedure was used for synthesis of PDMA-PHFBMA-PBzMA and PDMA-PBzMA-PHCFDDMA block copolymers.

### 2.2. Analysis and Characterization of Block Copolymers

Copolymer molar mass distributions were determined using size exclusion chromatography (SEC) performed with a double detector array from Viscotek (TDA 305, Malvern instruments, Worcestershire, UK). The Viscotek SEC apparatus was equipped with two mixed-columns (LT4000L, mixed low, Malvern Panalytical, Malvern, UK) with common particle size of 5 µm using THF as an eluent (1.0 mL/min). The Viscotek system contains a refractive index detector (RI, concentration detector), and a four-capillary differential viscometer. OmniSEC software (version 10, Malvern Panalytical, Malvern, UK) was used for data analysis and acquisition. The average molar mass (*M_n_*) and dispersity index (*Ð*) were calculated relative to polystyrene standards.

^1^H NMR spectra were acquired with a Bruker 300 MHz spectrometer (Bruker, Wissembourg, France) using CDCl_3_ or CD_2_Cl_2_.

Dynamic light scattering (DLS) measurements were conducted using a Malvern Instruments Zetasizer Nano series instrument (Malvern Panalytical, Malvern, UK) equipped with a 4 mW He-Ne laser operating at 633 nm, an avalanche photodiode detector with high quantum efficiency, and an ALV/LSE-5003 multiple tau digital correlator electronics system. Samples were prepared at 0.1% *w*/*w* by diluting the PISA suspension with ethanol and the scattered light was detected at 173°.

Transmission electron microscopy (TEM, JEOL Europe SAS, Croissy Sur Seine, France) studies were conducted using a JEOL 1200 EXII instrument operating at 120 kV equipped with a numerical camera. To prepare TEM samples, 5.0 μL of a dilute copolymer solution (0.1% *w*/*w*) was placed onto a carbon-coated copper grid (60 s), stained using an aqueous solution of ammonium molybdate 99.98% (20 s), and then dried under ambient conditions. The copolymers containing fluorinated methacrylate were not stained. ImageJ software was used for image analysis. The reported mean diameter measurements were performed on 100 nanoparticles.

## 3. Results and Discussion

### 3.1. RAFT Dispersion Polymerization of HFBMA in Ethanol

An ethanol soluble macro-CTA (PDMA) was synthesized via RAFT solution polymerization. The mean degree of polymerization (DP) of this macro-CTA was calculated to be 39 from the ^1^H NMR spectrum of a purified polymer sample (PDMA_39_, *M_n_* = 5100 gmol^−1^, *M_w_* = 5900 gmol^−1^, *Đ* = 1.16). This macro-CTA was then chain extended with semi-fluorinated monomers in ethanol at 70 °C via RAFT dispersion polymerization.

**Figure 1:**
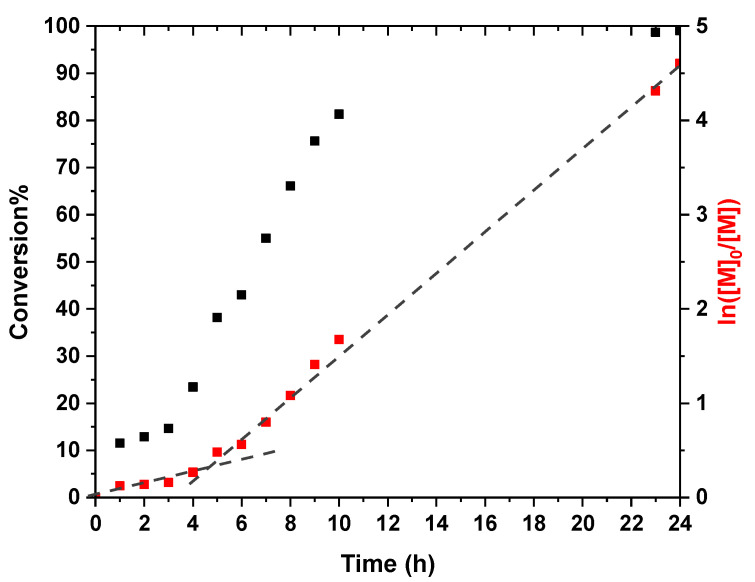
Kinetic data (Conversion %: Black squares, Semi-log: Red squares) obtained for the RAFT dispersion polymerization of HFBMA at 10% *w*/*w* solids in ethanol at 70 °C using a PDMA_39_ macro-CTA at a macro-CTA/AIBN molar ratio of 5/1. The targeted diblock composition was PDMA_39_–PHFBMA_200_.

**Figure 2:**
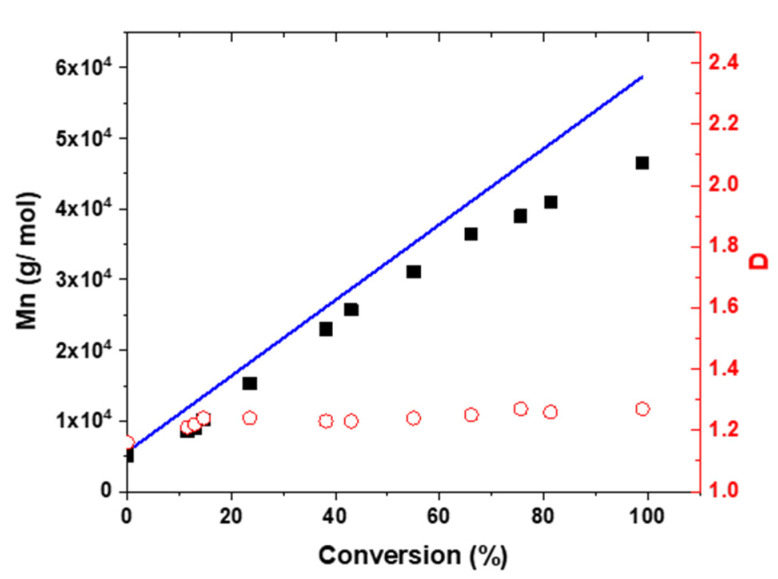
Evolution of number-average molar mass (*M_n_*, black squares) and dispersity (*Đ*, red open circles) with conversion for the RAFT dispersion polymerization of HFBMA at 10% *w*/*w* solids in ethanol at 70 °C using a PDMA_39_ macro-CTA with macro-CTA/AIBN molar ratio of 5/1. The targeted diblock composition was PDMA_39_–PHFBMA_200_.

A kinetic study of this chain extension with poly(heptafluorobutyl methacrylate) (PHFBMA) was conducted using a target DP of 200. Almost full conversion was obtained after 24 h of polymerization as judged by ^1^H NMR analysis (Table 1). The semi-logarithmic plot shows two distinct regimes: (1) An initial 4 h period where the rate of polymerization is relatively slow, and the remaining 16 h showing an increase in the polymerization rate (Figure 1). This rate enhancement as reported previously is attributed to the onset of micellar nucleation with the nascent monomer-swollen micelles acting as a nanoreactors [14]. Both rate regimes followed the first order kinetics law, as expected for a RAFT polymerization. The evolution of the molar mass of the copolymer with conversion was linear, confirming the control of the HFBMA polymerization (Figure 2). The good control of the RAFT polymerization was further confirmed by the low dispersities of the copolymer, which remained below 1.30 throughout the reaction (Figure 2).

**Figure 3:**
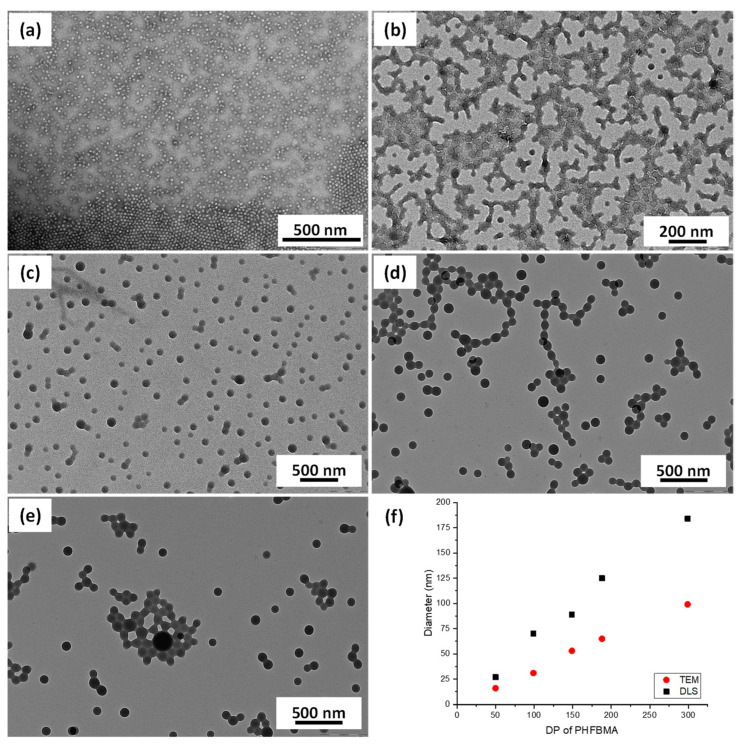
TEM images obtained for PDMA_39_–PHFBMA_x_ nanoobjects prepared at 10% *w*/*w* via RAFT alcoholic dispersion polymerization of HFBMA with (**a**) x = 50, (**b**) x = 99, (**c**) x = 149, (**d**) x = 188, and (**e**) x = 299. (**f**) Evolution of the average particles diameter measured by TEM (red dots) and DLS (black squares) with the DP of the PHFBMA core-forming block.

A series of PDMA_39_-PHFBMA_x_ diblock copolymers nano-objects were prepared at 10 *w*/*w* % with varying of the DP of the PHFBMA core-forming block between 50 and 300 (Entries 2–6, Table 1). In all cases near full HFBMA conversions were observed within 24 h. The DLS analysis of the resulting particles showed that the particle average hydrodynamic diameters increased linearly with the increasing length of the PHFBMA core-forming block (Table 1 and Figure 3f). TEM images of these particles showed that they were all spherical regardless of the DP of the PHFBMA block. This suggests that the PHFBMA chains did not have sufficient mobility to allow the re-organization into higher order morphologies.

**Scheme 1:**
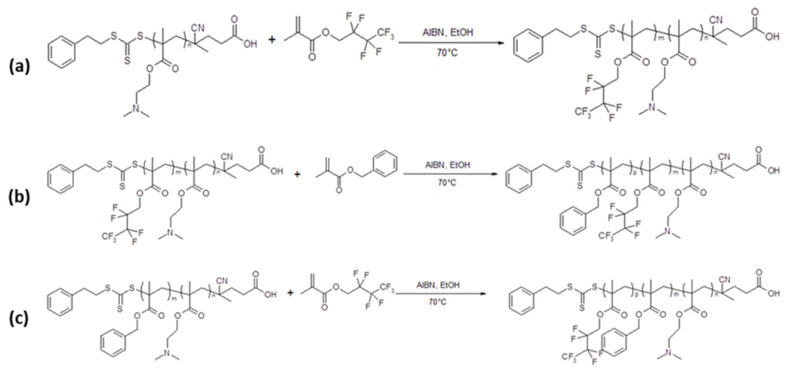
Sequential RAFT alcoholic dispersion polymerization at 70 °C in ethanol using a poly(2-(*N*,*N*,dimethylamino)ethyl methacrylate) as chain transfer agent of: (**a**) heptafluorobutyl methacrylate (HFBMA), (**b**) Benzyl methacrylate (BzMA), (**c**) heptafluorobutyl methacrylate (HFBMA), resulting in the formations of PDMA-PHFBA and PDMA-PHFBMA-PbzMA diblock copolymers ((**a**,**b**) respectively), and PDMA-PBzMA-PHFBMA triblock copolymers (**c**).

**Figure 4:**
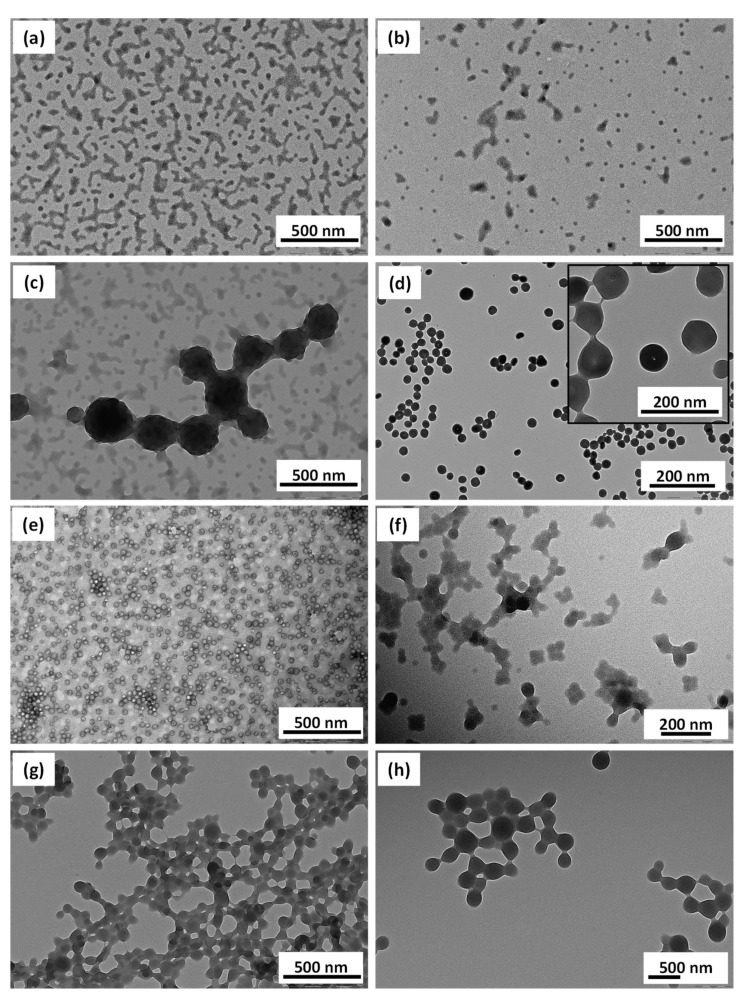
TEM images obtained for PDMA_39_–PHFBMA_x_- PBzMA_y_ nano objects prepared at 10% *w*/*w* with (**a**) x = 50, y = 69; (**b**) x = 99, y = 69; (**c**) x = 149, y = 69; (**d**) x = 199, y = 66; (**e**) x = 50, y = 198; (**f**) x = 99, y = 198; (**g**) x = 149, y = 198; (**h**) x = 199, y = 198.

To obtain other morphologies than spheres a second hydrophobic block of poly(benzyl methacrylate) (PBzMA) was added (Scheme 1b). Benzyl methacrylate was polymerized under seeded dispersion polymerization conditions at 10% *w*/*w* and 70 °C in the presence of the previously prepared PDMA_39_–PHFBMA_x_ diblock copolymer spheres (with x = 50, 99, 149 and 199). It should be pointed out that unlike our previously reported ABC block copolymers prepared by PISA, for which the addition of the semi-fluorinated monomer dissolved the PMAA-PBzMA diblock particles [12], here the addition of the BzMA did not dissolve the PDMA-PHFBMA spherical particles. The polymerization of the BzMA was carried out for 24 h resulting in quantitative monomer conversions (≥95%). In the first set of reactions, a PBzMA of DP = 70 (Entries 7–10, Table 1) was targeted as the third block. As shown in Figure 4a–d and Table 1 only spherical particles were obtained regardless of the length of the two core-forming blocks. This could be explained by the fact that the BzMA did not dissolve the PDMA-PHFBMA nanoparticles, leading to the observed frozen spherical morphologies. Nevertheless, the average diameters of the resulting ABC triblock copolymer spheres increased with the addition of the PBzMA_70_ block. Both hydrodynamic and dry diameters of the particles measured by DLS and TEM image analysis showed gradual increase from 60 to 375 nm (DLS) and from 28 to 225 nm (TEM). Increasing the DP target of the PBzMA third block to 200 (Entries 11–14, Table 1) did not promote the formation of higher order morphologies either (Figure 4e–h). However, a closer examination of the TEM images suggest that the particles have internal structure. PDMA_39_-PHFBMA_149_-PBzMA_69_ particles (Figure 4c), for example, seem framboidal and are reminiscent of the PGMA-PHPMA-PBzMA particles reported by Armes and coworkers [33] where the incompatibility between PHPMA and PBzMA resulted in the formation of raspberry-shaped particles. Increasing the length of the PHFBMA chains resulted in spherical particles with smoother surface (Figure 4e–h) although internal structure can still be seen. This is most probably due to the incompatibility of the two core-forming blocks (PBzMA and PHFBMA).

**Figure 5:**
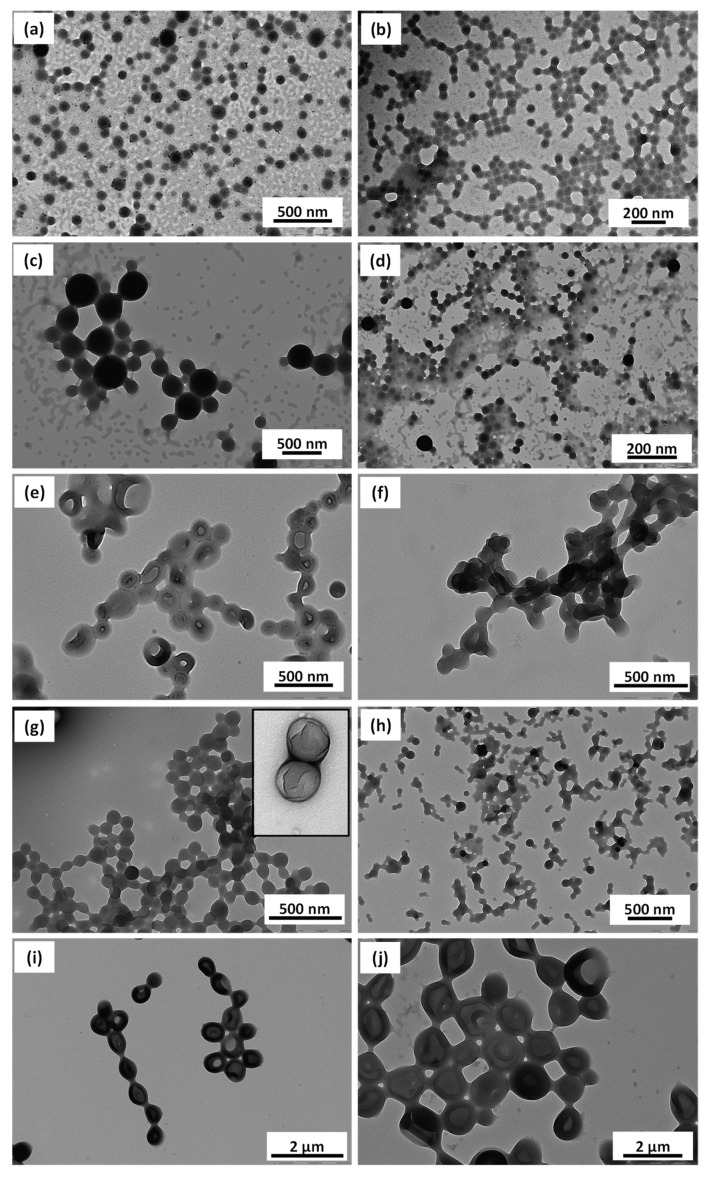
TEM images obtained for PDMA_39_-PBzMA_x_-PHFBMA_y_ nano objects prepared at 10% *w*/*w* with (**a**) x = 69, y = 50 (**b**) x = 69, y = 99 (**c**) x = 69, y = 149 (**d**) x = 69, y = 196 (**e**) x = 99, y = 65 (**f**) x = 149, y = 141 (**g**) x = 198, y = 50 (**h**) x = 198, y = 96 (**i**) x = 198, y = 144 (**j**) x = 198, y = 189.

In the next set of experiments the core-forming blocks were synthesized in the reverse order (PBzMA first, as the B block; and PHFBMA second, as the C block) to see if other morphologies than spheres could be obtained. PDMA_39_-PBzMA_69_ diblock copolymer spherical particles were first synthesized (Entry 16, Table 1). This sample was then divided in four portions that were used for the RAFT dispersion polymerization of HFBMA in ethanol targeting PHFBMA DPs of 50, 100, 150 and 200 (Entries 20–23, Table 1). Here, contrary to the previous set of ABC triblock copolymers where PHFBMA was the B block, the addition of HFBMA to the PDMA-PBzMA nanoparticles suspension partially dissolved the PBzMA block. In most cases, only spherical textured particles were formed (Figure 5a–d). However, both PDMA_39_-PBzMA_99_-PHFBMA_65_ and PDMA_39_-PBzMA_149_-PHFBMA_141_ triblock copolymers (Entries 24–25, Table 1) self-assembled into vesicles featuring very clear inner dark ring (Figure 5e,f). TEM samples of fluorinated block copolymer nano objects were prepared without staining; hence the dark ring indicates the region/position with high concentration of fluorine atoms. PDMA-PBzMA diblock copolymer vesicles with longer PBzMA were synthesized (PDMA_39_-PBzMA_198_). These vesicular dispersions were then used in the RAFT polymerization of HFBMA to produce triblock copolymer PDMA_39_-PBzMA_198_-PHFBMA_x_ with x = 50, 96, 144 and 189 (Entry 26–29, Table 1). The PHFBMA_50_—containing triblock self-assembled into spherical particles with a visible sheet-like structure partially covering the surface of the spherical particles (Figure 5g). Increasing the DP of PHFBMA to 96 resulted in the formation of fused spherical particles (Figure 5h). Addition of more HFBMA repeat units pushed the morphology towards vesicles possessing a distinctive dark ring within their membranes, indicating the PHFBMA rich zone (Figure 5i,j). The comparison of the self-assembled objects formed from ABC and ACB triblock copolymers of similar composition (Table 1, Entries 13–14) illustrates the importance of particle swelling and chain mobility for the evolution of the morphologies. Here, while BzMA could not dissolve the PHFBMA block, HFBMA did act as solvent for PBzMA (evidenced by the turbid diblock copolymer solution turning less turbid or even clear after addition of HFBMA).

**Figure 6:**
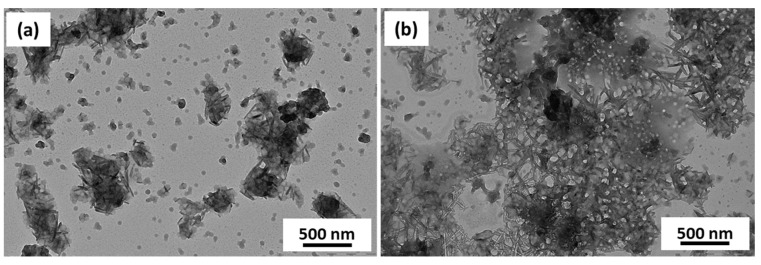
TEM images obtained for PDMA_58_–PBzMA_198_-PHCFDDMA_x_ prepared at 10% *w*/*w* with (**a**) x = 19 (**b**) x = 50.

To see the effect of increasing the number of fluorinated units on the phase separation of the two core forming blocks, HFBMA (containing 7 fluorine atoms) was replaced with HCFDDMA (containing 21 fluorine atoms and T_m_ of ~130 °C). A longer PDMA (DP = 58, *M_n_* = 6200 g/mol, *M_w_* = 7400 g/mol and *Đ* = 1.20) was used to increase the colloidal stability of the resulting nanoparticles. The diblock copolymer PDMA_58_-PBzMA_198_ formed spheres (Entry 1, Table S1). This diblock copolymer was used for the polymerization of HCFDDMA, targeting a DP of 20 (Entry 2, Table S1) that reached 95% conversion in 24 h (*M_n_* = 21,000 g/mol, *M_w_* = 53,000 g/mol and *Đ* = 1.29). The conversion for the DP = 50 polymerization could not be calculated as the sample was not soluble in any of the common organic solvents (or their mixtures) tried (Entry 3, Table S1). TEM images revealed that the PDMA_58_-PBzMA_198_-PHCFDDMA_19_ triblock copolymer formed spherical particles covered with needle-shaped structures (Figure 6a). PDMA_58_-PBzMA_198_-PHCFDDMA_50_ formed a mixture of spheres along with crystalline shards and rod like structures (Figure 6b). Such phase separations and rigid crystalline structures have been previously observed in the self-assembly of PVDF containing BCPs [34,35,36,37].

### 3.2. PDMA-PBzMA-PTDFOA

**Scheme 2:**
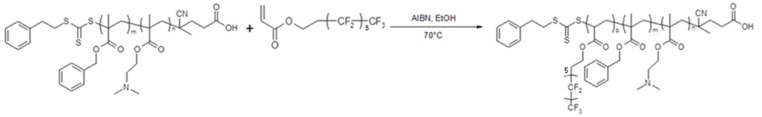
Synthesis of triblock copolymers prepared by RAFT dispersion polymerization of tridecafluorooctyl acrylate, TDFOA at 70 °C in ethanol using PDMA_58_–PBzMA_198_ spherical particles as macroCTA.

**Figure 7:**
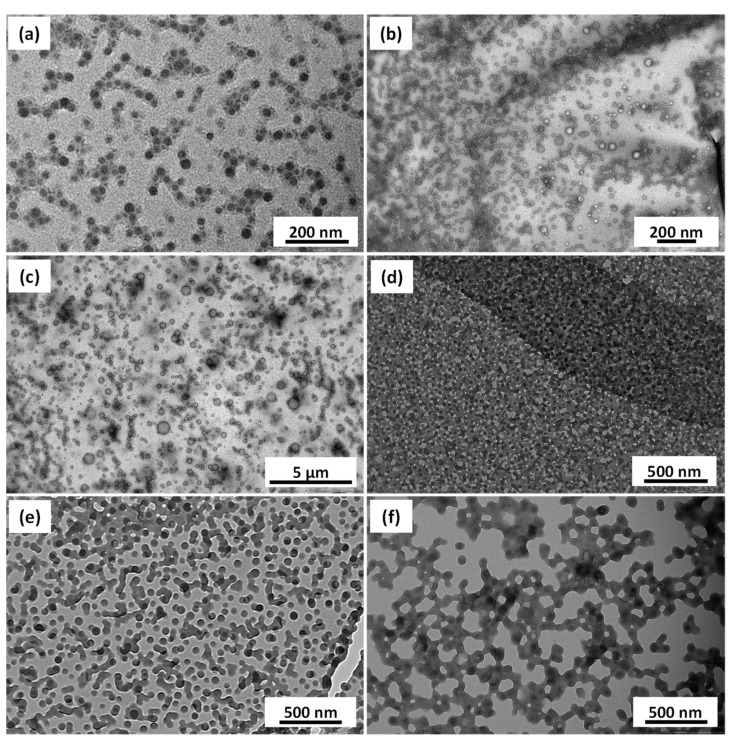
TEM images obtained for PDMA_58_-PBzMA_x_-PTDFOA_y_ self-assembled nano objects prepared at 10% *w*/*w* by RAFT dispersion polymerization of PTDFOA in ethanol, with (**a**) x = 69, y = 48 (**b**) x = 69, y = 91 (**c**) x = 69, y = 144 (**d**) x = 194, y = 44 (**e**) x = 194, y = 83 (**f**) x = 194, y = 120.

In order to see if a more flexible backbone would affect the morphology of the particles and their phase segregation, an acrylate monomer bearing 13 fluorine atoms (tridecafluorooctyl acrylate, TDFOA) was polymerized as the second core-forming block (Scheme 2). As summarized in Table S2, near complete conversions were obtained when the PDMA_58_-PBzMA_69_ was used as macro-CTA. Increasing the DP of PBzMA to 194 resulted in a drop in the conversion of TDFOA perhaps due to the unavailability of chain end-groups, since the crystalline TDFOA might not be able to swell the particle core well enough. TEM analysis (Figure 7) of the resulting ABC triblock copolymers revealed the formation of isolated and fused/connected spherical particles. Almost all images showed darker zones, indicating the fluorine rich blocks. Since these TEM samples were not stained the lighter halo is likely formed by the PBzMA chains while the intense dark central core is solely formed by the fluorinated PTDFOA chains.

## 4. Conclusions

In summary, a series of di and triblock copolymers containing semi-fluorinated monomers were synthesized via RAFT controlled PISA formulation in ethanol at 70 °C. The monitoring of the RAFT polymerization of heptafluorobutyl methacrylate (HFBMA) using a PDMA macro CTA showed that high conversions were achieved within 24 h and SEC analyses indicated relatively well-controlled polymerizations. TEM studies revealed well-defined spherical nanoparticles. To form higher order morphologies the synthesized diblock copolymers were chain extended with BzMA. However, due to the rigidity of the semi-fluorinated 2nd block the spherical particles grew in size but did not evolve to higher morphologies. The triblock copolymer analog featuring PBzMA as the second block however did result in the formation of spherical particles and vesicles showing micro-phase separation of the core-forming blocks. The triblocks formed using a semi-crystalline fluorinated monomer (heneicosafluorododecyl methacrylate (HCFDDMA)) led to formation of shard like structures often reported for the self-assembly of crystalline BCP. It was also demonstrated that using an acrylate back bone (tridecafluorooctyl acrylate (TDFOA)) instead of the methacrylate the evolution of particle morphology was not improved as only spheres or fused spheres where formed. The data presented here underline the importance of polymer chain mobility, compatibility and block order in the self-assembly of block copolymer under PISA process.

## Figures and Tables

**Table 1:** Summary of di- and triblock copolymers characterizations.

No.	Target Composition	Conversion%	Calculated DP *	Mean Diamater		Morphology
				DLS (nm)	TEM (nm)	
1	PDMA_50_	71	39			
2	PDMA_39_-PHFBMA_50_	99	50	27 (0.08)	16	Sphere
3	PDMA_39_-PHFBMA_100_	99	99	70 (0.04)	31	Sphere
4	PDMA_39_-PHFBMA_150_	99	149	89 (0.07)	53	Sphere
5	PDMA_39_-PHFBMA_200_	94	188	125 (0.08)	65	Sphere
6	PDMA_39_-PHFBMA_300_	99	299	184 (0.04)	99	Sphere
7	PDMA_39_-PHFBMA_50_-PBzMA_70_	99	69	60 (0.10)	28	Sphere
8	PDMA_39_-PHFBMA_99_-PBzMA_70_	99	69	70 (0.12)	48	Sphere
9	PDMA_39_-PHFBMA_149_-PBzMA_70_	99	69	80 (0.07)	224	Sphere
10	PDMA_39_-PHFBMA_199_-PBzMA_70_	94	66	375 (0.09)	213	Sphere
11	PDMA_39_-PHFBMA_50_-PBzMA_200_	99	198	400 (0.11)	30	Sphere
12	PDMA_39_-PHFBMA_99_-PBzMA_200_	99	198	1190 (0.13)	43	Sphere
13	PDMA_39_-PHFBMA_149_-PBzMA_200_	99	198	1247 (0.09)	68	Sphere
14	PDMA_39_-PHFBMA_199_-PBzMA_200_	99	198	1810 (0.10)	99	Sphere
15	PDMA_39_-PBzMA_60_	98	59	40 (0.05)	26	Sphere
16	PDMA_39_-PBzMA_70_	99	69	46 (0.02)	30	Sphere
17	PDMA_39_-PBzMA_85_	99	84	87 (0.07)	60	Sphere/Worm
18	PDMA_39_-PBzMA_120_	97	117	160 (0.07)	86	Worms/Vesicle
19	PDMA_39_-PBzMA_200_	99	198	325 (0.04)	204	Vesicle
20	PDMA_39_-PBzMA_69_-PHFBMA_50_	99	50	100 (0.12)	48	Sphere
21	PDMA_39_-PBzMA_69_-PHFBMA_100_	99	99	125 (0.08)	93	Sphere
22	PDMA_39_-PBzMA_69_-PHFBMA_150_	99	149	200 (0.011)	120	Sphere
23	PDMA_39_-PBzMA_69_-PHFBMA_200_	98	196	200 (0.14)	336	Sphere
24	PDMA_39_-PBzMA_99_-PHFBMA_100_	65	65	360 (0.07)	150	Vesicle
25	PDMA_39_-PBzMA_149_-PHFBMA_150_	94	141	860 (0.10)	230	Vesicle
26	PDMA_39_-PBzMA_198_-PHFBMA_50_	99	50	210 (0.06)	73	Sphere
27	PDMA_39_-PBzMA_198_-PHFBMA_100_	96	96	430 (0.14)	122	Fused Spheres
28	PDMA_39_-PBzMA_198_-PHFBMA_150_	96	144	600 (0.11)	145	Vesicle
29	PDMA_39_-PBzMA_198_-PHFBMA_200_	94	189	2200 (0.12)	304	Vesicle

* Calculated DP = Target DP × Conversion by ^1^H NMR.

## Data Availability

The data presented in this study are available on request from the corresponding author.

## Supplementary Materials

The following are available online at https://www.mdpi.com/article/10.3390/polym13152502/s1, Figure S1: ^1^H NMR spectra of unpurified (a) and purified (b) PDMA macro-CTA. Table S1: Summary of assemblies focusing on HCFDDMA, Table S2: Summary of the different copolymers synthesized and their dimensions, Table S3: Summary of selected copolymers molecular weights and dipersities obtained from THF SEC calibrated with PS standards.
