# Supplementary material for: Semi-Fluorinated Di and Triblock Copolymer Nano-Objects Prepared via RAFT Alcoholic Dispersion Polymerization (PISA)
Source: Polymers (Basel). 2021 Jul 29;13(15):2502. doi: 10.3390/polym13152502 (PMC8347566; doi:10.3390/polym13152502)

polymers

MDPI

Article

Supporting Information

Semi-fluorinated di and triblock copolymer nano-objects prepared via RAFT alcoholic dispersion polymerization (PISA)

Gregoire Desnos [1], Adrien Rubio [1], Chaimaa Gomri [1], Mathias Gravelle [1], Vincent Ladmiral [2] and Mona Semsarilar [1,*]

[1] IEM, Univ Montpellier, CNRS, ENSCM, Montpellier, France.

[2] ICGM, Univ Montpellier, CNRS, ENSCM, Montpellier, France.

Table S1: Summary of assemblies focusing on HCFDDMA

Nº	Composition	Target DP	Conversion %	Theo. DP, x	Size DLS (nm)	Size TEM (nm)
1	PDMA$_{58}$-PBzMA$_x$	200	99	198	70	46
2	PDMA$_{58}$-PBzMA$_{198}$-PHCFDDMA$_x$	20	95	19	172	60
3	PDMA$_{58}$-PBzMA$_{198}$-PHCFDDMA$_x$	50	NA	NA	NA	NA

Table S2: Summary of the different copolymers synthesized and their dimensions

Nº	Composition	Target DP	Conversion %	Theo. DP, x	Size DLS (nm)	Size TEM (nm)	Morphology
1	PDMA$_{58}$-PBzMA$_x$	70	99	69	40	23	Sphere
2		200	97	194	75	46	Sphere
3	PDMA$_{58}$-PBzMA$_{69}$-PTDFOA$_x$	50	95	48	58	26	Sphere
4		100	91	91	95	49	Sphere
5		150	96	144	160	54	Sphere
6	PDMA$_{58}$-PBzMA$_{194}$-PTDFOA$_x$	50	88	44	70	52	Sphere
7		100	83	83	90	61	Sphere
8		150	79	120	170	69	Sphere

Table S3: Summary of selected copolymers molecular weights and dipersities obtained from THF SEC calibrated with PS standards.

Nº	Composition	M_n (g/mol)	M_w (g/mol)	Đ
1	PDMA$_{39}$-PBzMA$_{198}$-PHFBMA$_{96}$	37000	45000	1.22
2	PDMA$_{39}$-PBzMA$_{198}$-PHFBMA$_{144}$	59000	85000	1.45
3	PDMA$_{39}$-PHFBMA$_{99}$-PBzMA$_{198}$	98000	126000	1.28
4	PDMA$_{39}$-PHFBMA$_{149}$-PBzMA$_{198}$	100000	123000	1.23
5	PDMA$_{58}$-PBzMA$_{194}$-PTDFOA$_{83}$	64000	79000	1.23
6	PDMA$_{58}$-PBzMA$_{194}$-PTDFOA$_{120}$	42000	52000	1.25

Figure S1. 1H NMR spectra of unpurified (a) and purified (b) PDMA macro-CTA. For more details please refer to entry 1 in Table 1.

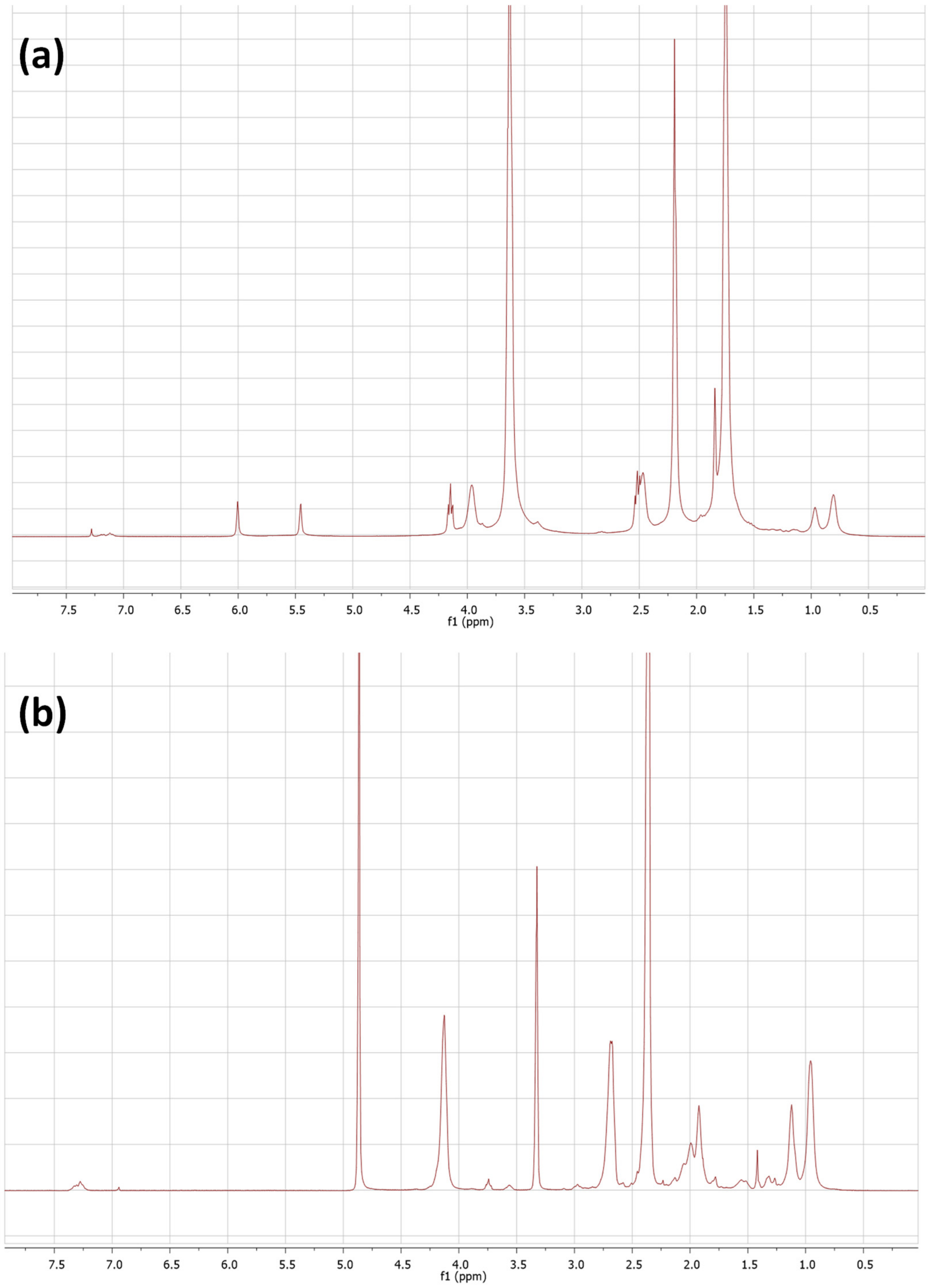

Supplement: Supplementary file 1 [file polymers-13-02502-s001.zip › polymers-1306125-supplementary.pdf]
